# St. Augustine Meeting—Abstracts

**Published:** 1898-05

**Authors:** J. M. Walker


### THE DENTAL REGISTER.

VOL. LII.] MAY, 1898. [No. 5.

### PROCEEDINGS.

### St. Augustine Meeting—Abstracts.

Reported for the DENTAL REGISTER by Mrs. J. M. Walker.

DENTAL EDUCATION.

DR. J. PERCY CORLEY, Greensboro, Ala., read a paper entitled "The Educational Features of the Dental Association."

The accomplishment of a profession is limited only by its standard of education. The three great educational factors are the journal, the college and the association.

The dental journal is certainly the vanguard in our march of progress. Our dental literature has contributed more to the advancement of the profession, scientifically, than any other branch of the educational trio. It is an outgrowth of its influence that we have the college and the association. The colleges embody in their curriculum the foundation for both a technical and a scientific education. The college professor should be an artist with instinctive genius for discerning the scattered forces of our nature and arranging them in harmonious accord. There is about the ideal college professor a personal magnetism, a kind of hero worship which touches the hidden springs of the student's soul and wakes into life and activity all the hidden possibilities of life. The college has a direct bearing upon the *personnel* of the profession. As I folded the much-prized parchment and passed out of the college doors into the world of action, I asked one of my professors for a parting benediction. He gave it in these words: "Attend the Associations." I could but poorly appreciate its value then, but to-day I realize that it is the most worthy advice I ever received. The Association is a great confessional where we recount our failures, to which we bring our problems and also the treasures which we have gathered; it is a great storehouse holding within its capacious vaults the accumulated wealth of hundreds of hours of study and toil. It is a kind of professional Mecca to which we make annual pilgrimages to pay tribute at the shrine of progress, and to acquire strength to bear the ever-increasing responsibilities of professional life. There ought to be a greater premium upon Association work; the public ought to require that every practitioner attend the meetings of his Association. May this convention mark the dawn of a new era in dentistry which shall spread as the morning light until the dark shadows of ignorance and superstition shall have given place to the glory of intellectual day. The social, intellectual and clinical features of the "Southern" have stood for years without a parallel. Let us, then, build upon the prestige of the past a "Parthenon" for the future.

DISCUSSION.

In the discussion of this paper, Dr. H. N. Johnson said that it was an education in itself. He spoke of the high standing that American dentistry had hitherto attained, and the necessity of keeping up to that standard in order to maintain our standing in foreign countries. He spoke of the gratifying fact that raising the standard of dental college education and increasing the number and length of terms, instead of deterring students, had seemed to actually increase the number of matriculants, with a gratifying improvement in the character and capacity of the applicants. He spoke of the comparatively larger clientele necessary to maintain the dentist than in the other professions. A patient whose work has been thoroughly done may not require your services again for a year, while many patients may require the services of the physician every week in the year. Many people never seek the services of the dentist; the same can not be said of the doctor. A lawyer may receive many thousand dollars as a fee in a single case—can that be said of the dentist?

Dr. B. Holly Smith did not agree with Dr. Johnson as to the number of patients necessary to keep a dentist employed, or that work once done would not require doing over again. He thought there was a necessity for dentists who would be satisfied to emulate the country doctor with his small but sure practice—there are so many people who have to ride four or five miles to seek the services of a dentist; there is need for dentists who will fill these vacant places and place the benefits of dental services within the reach of those far from the great cities. The country is not overcrowded, though the large cities may be.

Dr. J. Y. Crawford said that he wanted to endorse every word of the paper as being the truth, the whole truth, and nothing but the truth.

Dr. D. R. Stubblefield, Nashville, Tenn., contributed a paper on "Dental Literature," giving what he termed a heretical view of the subject. The nature and essence of dentistry limits its literature. Dentistry demands of its followers manual labor; it inheres in the very nature of our calling. There will never be tooth-filling machines that do not stand on human feet and use human hands. This very fact must limit us somewhere, somehow. A large proportion of those who embrace our specialty are not the most highly cultivated in mind; they can not understand the abstract. Our literature must be illustrated to reach the average intelligence. We appreciate what we *see* better than what we hear. None but money-making concerns can publish journals which demand illustration, both new and original, for every issue. It demands money, and lots of it. Dentists are not able to have a literature if they depend upon their own resources. Let us, therefore, hold up the hands of those who come to our rescue. If the journals are not as good as we would like them to be, it is because we do not furnish the material. They simply print what we utter. The publishers call for bread, and you give them a stone; you expect brick, and you give no straw. If the advertisements offend our esthetic taste, we can keep our eyes off them; they may have a vital purpose to fulfill. We are too sensitive entirely, under the circumstances.

Dr. D. E. Wilm, Washington City, contributed an excellent and impartial review of the journalism of the past year, and of the valuable text-books which have been published during the year.

Dr. W. W. H. Thackston, in a paper entitled The "Prostitution and Abasement of Dental Practice," dealt in scathing terms with the degradation of the college degree and the diploma, as advertised by the dental parlors of the day—"Only Graduate Assistants Employed," and suggested as the remedy that all diplomas and degrees be made revocable by the college issuing them—such revocation to be published in the annual announcements of the respective colleges and in the secular press of the place of residence of such offender. Only by this, or some similar method, can we hope to abate the reproach that is being brought upon our educational institutions. The time has come for the "brakes" to be put on and set hard upon the traitorous graduates of the dental colleges.

Dr. W. E. Walker, Chairman of the Committee on Dental Hygiene, read a report, reviewing the dental literature of the past year in its bearing upon the subject of Hygiene—the responsibility of the dentist for the hygiene of the oral cavity; as a teacher of the public in this direction; the work of the bacteriologists in developing a system of prophylaxis. He quoted from a paper by Dr. M. V. Ball, who characterizes dental caries as a wound which may serve as a focus of disease, decaying teeth being "so many little test tubes of microbe cultures ready to inoculate the human body whenever and wherever its natural resistance is lowered." He quoted from a paper by Dr. J. H. Kellogg on the relations existing between decaying teeth and functional disorders of the stomach.

Dr. R. R. Andrews, in his address as Chairman of the Section in Stomatology of the American Dental Association, spoke of the connection between bacterial growths in the oral cavity and reverse disturbance of the general health, and referred to an article by Stark on the connection between carious teeth and tuberculous cervical glands. Among other papers cited by Dr. Walker in this review is one entitled "Bacteria of the Mouth," by Dr. A. C. Hart, of San Francisco, who, in regard to dental decay, offers a theory of such treatment as shall so harden the inter-cellular cement substance of the teeth as to enable them to resist the solvent action of bacteria.

The editor of the *Dental News* urges the importance of a closer study of oral hygiene by the physician, as by a broader knowledge on this subject many of the diseases common to the alimentary tract could be aborted or lessened in intensity.

Dr. A. W. Harlan, in a paper entitled "The Drug Habit," cautions against the excessive use of "washes, lotions, pastes and powders" for oral cleansing, claiming that the continued use of these agents not only blunts the sensibilitty of he mucous membrane, but tends to the fixing of "a drug habit" capable of doing a vast amount of injury.

As a measure of hygienic precaution in preventing the X-ray burns of which numerous instances were published during the past year, Dr. Walker quoted from Dr. C. L. Leonard, who mentioned as a fact, recently made known, that a sheet of aluminum, if grounded and placed between the tube and the patient, will prevent the burn, while interfering in no way with the X-ray phenomenon.

In the discussion of this paper, the subject of cleaning the teeth received much attention. Dr. Cowardin spoke of the difficulty of enforcing among patients the rules that we know to be efficacious. Dr. Chas. Sill tells them that if time is very limited, to devote to the teeth the last five minutes before retiring is the better time.

Dr. J. Y. Crawford endorsed Dr. Harlan's idea of blunting the sensibility of the mucous membrane by the use of mouth washes, and advocated very strongly the unlimited use of pure water as the best dentrifice—God Almighty's own prescription.

Dr. Walker spoke of the importance of instructing patients at the chair, and cited the case of a lady who had been under treatment for Rigg's disease for a long time. When she came into Dr. Walker's hands he found that the source of the trouble was simply impacted food between the teeth, of the presence of which she was not aware, the gums having become tolerant of its presence. Finding this condition repeated on the occasion of several visits, he apologized for his frankness and showed her the cause of the trouble and told her that the remedy lay in her own hands, but that treatment would be of no avail as long as that condition was renewed after every meal. She appreciated the information and profited by it. Dr. Walker has secured good results by instructing his patients to sit down while cleaning the teeth in order not to hurry through with it.

Papers on "Oral Hygiene" and the "Germ Theory of Dental Decay," by DR. S. W. FOSTER, of Atlanta, and DR. J. W. DAVID, of Mexia, Texas, were read.

DR. W. K. FORT, of Atlanta, read a paper on "Bacteriology," illustrated by a number of culture tubes showing the growth of microbes from an infected broach, from decayed dentine, etc., and also the inhibitory powers of various antiseptics, essential oils, etc., and also of different filling materials.

In the discussion of this paper, the danger of infection from septic instruments, and the necessity of thorough sterilization of all instruments and appliances used in operative dentistry was very fully discussed, in view of the liability of contaminating patients by carrying disease germs from one mouth to another.

DR. B. H. SMITH spoke of the efficacy of the vapor of formal, and described a simple apparatus for utilizing this disinfectant agent.

DR. W. E. WALKER described the aseptic cover for the bracket table used in his office—a sheet of rubber dam which is removed, together with all the instruments used for any patient, and with them disinfected by boiling for five minutes in water to which soda has been added, the addition of soda raising the temperature of boiling water, and thus increasing its germicidal powers. Repeated boiling does not injure the rubber dam in any way—a sample shown which had, as a test, been subjected to boiling over thirty times, being as strong and elastic as a piece of new rubber dam. The color is agreeable and does not reflect the light; it is noiseless and does not absorb the warmth from steel instruments, is not injured by acids, and being removed and sterilized, together with the instruments, at each change of patients, obviates all danger of contamination. For sterilizing the hands, Dr. Walker uses a soap which has been reduced to a jelly by grating finely and digesting in water, which forms an almost instantaneous lather all over the hands, to which is added a small portion of powdered mustard, which produces a pale, yellow, creamy lather of strong disinfectant power and leaves the hands feeling delightfully clean. For sterilizing instruments by boiling in soda water, Dr. Walker described an inexpensive arrangement—a large "agate ironware" pot with cover, in which is submerged a perforated tin vessel holding the instruments. Hand-pieces are subjected to the boiling, and then laid in a vessel of alcohol which prevents any danger of rust and cleanses them very perfectly. Mouth mirrors are laid in a solution of hydronaphthol. Napkins should be subjected first to steam heat, and then to dry heat after coming from the laundry, where they are liable to contagion after having been ironed.

Dr. B. Holly Smith recommends passing all broaches through the flame, and then dropping into the 40 percent formaldehyde. He also uses a 25 percent alcoholic solution of hydronaphthol. He spoke of the importance of being able to diagnose specific infection. For such cases he operates in sterilized lisle-thread gloves, reserving a special set of instruments to be used for no other patients.

Dr. Walker quoted statistics showing that one person in every fourteen in the United States has specific infection, but does not think that we can diagnose it in every case. Many are under the care of the physician who keeps down the symptoms, but for that very reason it is necessary to be all the more guarded against the danger of infection from unsuspected sources, and to be thorough in sterilizing all instruments used about the mouth.

The papers on "Hygiene," by Dr. S. W. Foster and Dr. J. W. David were discussed on the same general line.

The subject of the Appointment of Dental Surgeons in the Army and Navy was discussed in connection with a resolution offered by Dr. Chapple, of Atlanta, and a committee of five, Drs. J. A. Chapple, E. P. Beadles, B. Holly Smith, M. F. Finley and H. B. Noble, was appointed to bring this matter before Congress.

At the night session Wednesday, Dr. T. B. Hinman gave an interesting X-ray exhibit, and Dr. G. W. WELD gave a lecture with stereopticon exhibit, on the chemico-metallic method of filling root-canals.

A communication from DR. J. V. HALLER, of Virginia, was read, expressing the opinion that the results in root-canals treated by DR. WELD'S method are due to the chlorine which is liberated, the dry chlorine gas combining with hydrogen and liberating the oxygen which does the work in the canal of disinfecting and deodorizing. Silver nitrate is also thrown down if enough of the dental nitro-hydrochloric acid is employed.

In the discussion of the subject of root-canal filling, DR. CRAWFORD said that the difficulties of treating pulpless teeth and filling root-canals was not such a terrible bugbear as it seemed to be regarded by some. He does not think that the better class of practitioners average over 5 percent of failures. He thinks that when there is a slight soreness after filling the canals, the result is most apt to be favorable, an infiltrating action closing the foramen and rendering it impervious, septic emanations from the dentinal tubuli being of minor importance unless of sufficient force to uncork the foramen or even to burst the tooth, as has occurred. Nature's processes will close the apical foramen unless interfered with, as by a broach passed through the foramen carrying morbid matter, or a bubble of air entangled between the apex and the filling, or a wad of cotton lodged near the apex, attracting fluids from the outside.

DR. L. G. NOEL thinks that when the canals are small and difficult of access in badly-decayed molars it is better to cut off the crown until a solid foundation is reached upon which to place a good artificial crown over well-filled roots that have been made accessible by removal of the natural crown.

---

Under the head of "Prosthetic Dentistry," DR. C. L. ALEXANDER read a valuable paper embodying what he termed hints on crown and bridge-work. To attain perfection in this department all work must be constructed with a view to perfect hygienic relations, as well as esthetic appearance. All preliminary work on the teeth must be thoroughly done; the gums restored to health and dead teeth treated and filled. When shell crowns are placed either as individual crowns or as abutments, the work should be a perfect fit extending well down under the gum margin excluding the possibility of further decay. Where porcelain facings are to be used, it is not necessary to band the root. By beveling the edge of the root, gold plate can be burnished down which, when united to the crown proper, makes a very strong attachment. Cast abutments, instead of open-face band attachments, enable you to avoid the display of gold—a horrible deformity. Many failures in crown and bridge-work arise from having the gold for band and cusps too thick, making the work clumsy. The typical self-cleansing space adds to the durability of the work and is appreciated from a hygienic standpoint.

Dr. W. E. Walker described what he called an esthetic and hygienic crown—a method of mounting the Logan crown, with an inner band set in a groove between the mouth of the root-canal and the periphery. The band is ground level with the face of the root and soldered to a plate of gold or platinum, which is burnished to the face of the root and perforated for the pin of the Logan crown. The porcelain crown is beveled lingually, leaving a space of about the sixteenth of an inch at the lingual surface, and a plate of gold burnished to the face of the platinum. Around the pin is placed a mass of Parr's prepared wax. The plate, with the band in the groove, is warmed by throwing a current of warm air on it. The crown is also warmed and placed in position, the warmth causing the fluxed wax to adhere to both plates so that when cooled all can be removed together. When cool it is removed, the surplus wax trimmed away and the whole invested in Brown's Investment Fiber. If this is mixed with alcohol it can be set on fire, drying out the investment while the solder is being prepared. The space that was occupied by the wax is now filled with 18 or 20 caret gold solder, forming a crown which has the beauty and strength of the Logan crown, with advantage of a band for strength, while obviating the necessity of trimming the root and fitting an outside band which is liable to irritate the tissue and cause recession of the gum, exposing the gold. Though this crown is not described in Evans' Crown and Bridge-work, nor in the American Text-book of Prosthetic Dentistry, Dr. Walker found, on looking through the older journals, that a similar "inner band" was devised by Dr. Henry Maasch, and illustrated in the *Dental Cosmos* of March, 1889. The wedge-shaped section of gold between the porcelain and the root is similar to Dr. Gordon White's method of mounting the Logan crown. It is also a modification of the Hollingsworth method, but without the external band.

In the discussion of this subject, Drs. Cowardin and Holland and Alexander were very pronounced in opposition to the collar crown.

Dr. Cowardin believes they cause far more harm than the root-splitting, which is the only pretext for the use of a band. Dr. Cowardin uses the English tube crown, which takes a fine polish after grinding wherever necessary, and is of the same color all through. The pin being half-round the crown always goes to the same position, and being all of the same size repairs are easy if the porcelain breaks.

Dr. Crawford said that in condemning the collar crown, violence is done to one of the most beautiful and most successful operations in dentistry. From the standpoint of prosthetics he places the Richmond crown at the head of the list in point of durability and of beauty. If a crown has no collar, the joint being exposed to the fluids of the mouth, the root will disintegrate sooner or later.

Dr. Frank Holland was unsparing in his condemnation of the collar crown. That though he has seen many banded teeth, and has removed many collar crowns, he has never yet seen a banded anterior tooth that was clean and healthy. The highest order of dentistry is the closest approximation to nature, and nature will not tolerate any interference with the gum margins.

Dr. Walker would avoid extremes in either direction. There are cases where a band under the gum margin is indispensable, but they should be avoided whenever it is possible. If they are unhealthy for the anterior teeth they are equally so for the posterior teeth, and for single crowns can usually be dispensed with by using the Logan crown. For bridge abutments we must have the collar for strength.

Dr. H. H. Johnson emphasized the pin as long as possible to avoid leverage and consequent splitting of the root.

Drs. I. Simpson and A. A. Pearson advocate the collar crown.

Dr. S. Ewing Smith spoke of the necessity of reversing the slant of the root, trimming it so that it would be smaller at the edge of the face, so that the farther you drive the band on the tighter it will fit.

CHAPIN A. HARRIS MEMORIAL.

Dr. W. E. Walker, in a short paper, recalled the various attempts that have been made to accumulate a fund for the purpose of erecting a monument to the memory of Chapin A. Harris, the founder of the first dental college in the world, the editor of the first journal devoted to dental literature, the author of several standard text-books. He mentioned the work started by the Baltimore Association, reported by Dr. B. Holly Smith at the meeting of the Southern Dental Association in 1894; the work done by the Virginia State Society, and the committee appointed in 1894 by the Southern Association to further the work, with the co-operation of the different State Societies.

Dr. Beadles stated that, as instructed at that time, he had written to the secretaries of the different State Societies, with instructions to forward any amounts that might be raised to Dr. H. E. Beach, Clarksville, Tenn., who was made treasurer of the fund.

Dr. B. Holly Smith stated that the sum raised by the Baltimore Association was still in existence, and doubtless available.

After some discussion of the matter, on motion of Dr. Crawford; Dr. Walker was appointed a committee of one to bring the matter before the National Dental Association at its coming meeting when there would be a large crowd, and he had no doubt but that a sum could be raised then and there sufficient to erect a monument worthy of the name of Chapin A. Harris.

Two papers on Chemistry were read—one by Dr. B. Rut-

LEDGE, Chairman of the Committee, and one by DR. CHAS. A. BLAND.

DR. RUTLEDGE reviewed briefly the beginnings of chemistry in the mysteries of alchemy, the search for the philosopher's stone and the art of making gold, and its gradual development into the science of to-day. The importance of a knowledge of chemistry can not be over-estimated. It has a place in all the developments of life. In commerce, in art, in manufactures, in the trades and in the professions. Even the theologian finds in it a rich and fruitful field for illustrations. A knowledge of chemistry is one of the practical requirements of the successful dentist. In the selection of filling materials he must be able to judge of their chemical properties, color, density, conducting power, malleability, plasticity, etc. What more convincing argument in proof of chemical decomposition than the gases emanating from an improperly-constructed artificial denture or piece of bridge-work, with its retained vitiated secretions? Many of the most valuable remedial agents in use in dental practice are the product of the chemical laboratory. With a more general knowledge of this science, the secret nostrums of the quack would soon become a thing of the past.

As practical work in chemistry for the dental student, DR. BLAND enumerated the study of the metals and their compounds; the alloys, such as solders and amalgums; the analysis of the oral secretions and their reaction upon filling materials, especially the cements; an analysis of tooth substance in order to have a knowledge of the necessary food substances for building up poorly-calcified dental organs. The chemistry of caries is all-important, and this includes a knowledge of bacteriology. Antiseptics and disinfectants should be studied chemically—a knowledge of chemistry is necessary to successfully bleach teeth. In short, the field for chemical investigation is limitless in its practical application to dental science. With a chemical affinity for knowledge and a determination as indestructible as matter itself, success will crown our efforts.

A WINEGLASS of vinegar, it is said, will sober a very drunk person in twenty minutes.

